# Depression and Anxiety in Heart Transplant Recipients: Prevalence and Impact on Post-Transplant Outcomes

**DOI:** 10.3390/jpm13050844

**Published:** 2023-05-17

**Authors:** Emyal Alyaydin, Juergen Reinhard Sindermann, Jeanette Köppe, Joachim Gerss, Patrik Dröge, Thomas Ruhnke, Christian Günster, Holger Reinecke, Jannik Feld

**Affiliations:** 1 Department of Cardiology I—Coronary and Peripheral Vascular Disease, Heart Failure, University Hospital Muenster, Albert Schweitzer Campus 1, A1, 48149 Muenster, Germany; 2 Institute of Biostatistics and Clinical Research, University of Muenster, 48149 Muenster, Germany; 3 AOK Research Institute (WIdO), 10178 Berlin, Germany

**Keywords:** depression, anxiety, orthotopic heart transplantation, in-hospital mortality, survival

## Abstract

Background: Depression and anxiety (DA) are common mental disorders in patients with chronic diseases, but the research regarding their prevalence in heart transplantation (HTx) is still limited. Methods: We performed an analysis of the prevalence and prognostic relevance of DA in patients who underwent HTx between 2010 and 2018 in Germany. Data were obtained from Allgemeine Ortskrankenkasse (AOK), which is the largest public health insurance provider. Results: Overall, 694 patients were identified. More than a third of them were diagnosed with DA before undergoing HTx (*n* = 260, 37.5%). Patients with DA more often had an ischaemic cardiomyopathy (*p* < 0.001) and a history of previous myocardial infarction (*p* = 0.001) or stroke (*p* = 0.002). The prevalence of hypertension (*p* < 0.001), diabetes (*p* = 0.004), dyslipidaemia (*p* < 0.001) and chronic kidney disease (*p* = 0.003) was higher amongst transplant recipients with DA. Patients with DA were more likely to suffer an ischaemic stroke (*p* < 0.001) or haemorrhagic stroke (*p* = 0.032), or develop septicaemia (*p* = 0.050) during hospitalisation for HTx. Our analysis found no significant differences between the groups with respect to in-hospital mortality. The female sex and mechanical circulatory support were associated with an inferior prognosis. Pretransplant non-ischaemic cardiomyopathy was related to a favourable outcome. Conclusions: DA affect up to a third of the population undergoing HTx, with a greater prevalence in patients with comorbidities. DA are associated with a higher incidence of stroke and septicaemia after HTx.

## 1. Introduction

Depression and anxiety (DA) are protean disorders with a wide range of symptom severity and an increasing prevalence worldwide. According to recent reports, each condition alone affects approximately 4% of the global population [1]. DA prevalence is much higher in patients suffering from chronic diseases [2]. To date, studies indicate that roughly one-fifth of the patients diagnosed with heart failure (HF) also exhibit DA. This disease combination has been linked to an overall poor prognosis and lower quality of life (QoL) [3,4]. Nevertheless, patients with advanced HF who are awaiting heart transplantation (HTx) have to carry an even greater emotional burden, resulting in frequent recurrences and more severe symptoms of DA. Thus, it is to be expected that DA prevalence is higher in patients undergoing orthotopic HTx. In addition to a poor QoL, DA have been associated with limited compliance to recommended treatments and behaviours. All these factors may have deleterious consequences for the graft function in transplantation. A previous retrospective single-centre analysis reported an increased rate of hospitalisations and infectious complications after HTx in patients with DA [5]. The cardiovascular side effects of antidepressants are another factor limiting the treatment alternatives in most cases to selective serotonin reuptake inhibitors (SSRIs) only. Thus, the guidelines for the care of heart transplant recipients recommend regular evaluation of the mental health of patients undergoing HTx [6].

The aim of our study was to assess the prevalence and prognostic relevance of DA in patients undergoing orthotopic HTx.

## 2. Materials and Methods

### 2.1. Data Retrieval

Our study comprises data on adult heart transplant recipients anonymously retrieved from Allgemeine Ortskrankenkasse (AOK), which is an alliance of eleven regional funds covering up to a third of the population in Germany. According to recent reports, AOK provided insurance coverage for 25.938.841 affiliated persons and family members in the year 2018. In contrast to the private sector, public health insurance in Germany does not depend on gross income, health status, region, profession or age.

The timeline of our analysis was based on the index hospitalisation for orthotopic heart transplantation (HTx) (operation and procedure code 5-375.0). The study population was preselected among all subjects hospitalised for heart failure, coronary artery disease, myocardial infarction, peripheral artery disease, stroke and patients who underwent coronary or peripheral artery interventions in either an ambulant or stationary setting between 2008 and 2018.

The study included 694 adult patients who were insured by AOK and underwent HTx between 1 January 2010 and 31 December 2018. Baseline patient comorbidities were assessed using data from the two years that preceded the index hospitalisation. Follow-up data were collected until 31 December 2019. The median (interquartile range (IQR)) follow-up was 5.9 (IQR: 5.0) years. The information retrieved included primary, secondary and tertiary care results that were analysed in a coded manner. Diagnoses were documented according to the 10th Revision of the German Modification of International Classification of Diseases (ICD-10-GM). The diagnostic and therapeutic procedures performed were encoded using the German Modification of the Operation and Procedure Classification System (OPS). Anatomical Therapeutic Chemical codes (ATC-codes) were used for the prescribed medical treatment.

Data sampling was approved by the Ethics Committee of the Landesaerztekammer Westfalen-Lippe and the Medical Faculty of the University of Muenster (2019-212-f-S). 

### 2.2. Patient Population

We initially identified 852 patients who were hospitalised for orthotopic HTx (OPS: 5-375.0) between 1 January 2010 and 31 December 2018. Of these, 39 patients were excluded because they were <18 years of age at the time of the HTx procedure. In addition, 119 patients were excluded because of incomplete data. The study population was stratified into two groups according to whether a diagnosis of depression or anxiety was made prior to HTx (ICD-10-GM-Code: F30–F39 and F41) or not. We did not have access to detailed information regarding the methods implemented for the psychological assessment of the patients.

The primary outcome of our analyses was in-hospital mortality. The secondary endpoints included major adverse cardiovascular events, new onset or relapse of DA during the period covered by the study, and overall survival. Since, in most cases, both depression and anxiety are present simultaneously, and notably, most of the patients with depression have concurrent anxiety, we considered these conditions as a composite entity [1].

### 2.3. Statistics

The assessment of the impact of DA on the outcome of patients who had undergone HTx was performed using binary multivariable logistic regression models, which comprised major comorbidities and cardiovascular risk factors. Odds ratios with unadjusted 95% confidence intervals (CIs) for all features are shown in the graphs. Survival analyses were conducted using Kaplan–Meier estimators. Medication rates 90 days after index hospitalisation were estimated using competing risk models by calculating the cumulative incidence, where death was considered as a competing risk.

Quantitative data are reported as medians (IQRs) and compared using a two-sided Wilcoxon test. Qualitative data are presented as absolute values (percentages) and were compared using a two-sided chi-squared test. 

All *p*-values of the test procedures described above are purely descriptive and unadjusted. Inferential statistics are intended to be exploratory (hypotheses-generating), not confirmatory, and are interpreted accordingly.

Statistical analyses were performed using R version 4.0.2.

## 3. Results

### 3.1. Patient Characteristics

A total of 694 patients who underwent HTx from 2010 to 2018, the period covered by our study, were evaluated. More than a third of this population had documented diagnoses of DA prior to HTx (*n* = 260, 37.5%). We observed no statistically noticeable intergroup disparities in age, sex or previous mechanical circulatory support (MCS). Whilst the prevalence of cerebrovascular (CVD) and peripheral arterial disease (PAD) did not differ between the two groups, antecedent cardiac disease of ischaemic origin was more frequent amongst those diagnosed with DA (*n* = 276, 63.6% vs. *n* = 200, 76.9%; *p* < 0.001). Additionally, patients assigned to the DA group were more likely to have had a previous myocardial infarction (*n* = 129, 29.7% vs. *n* = 108, 41.5%; *p* = 0.001), percutaneous coronary interventions (*n* = 54, 12.4% vs. *n* = 51, 19.6%; *p* = 0.011) or stroke (*n* = 54, 12.4% vs. *n* = 55, 21.2%; *p* = 0.002). A closer look at the evaluable cardiovascular risk factors (CVRFs) and comorbidities revealed differences in the prevalence of hypertension (*n* = 356, 82.0% vs. *n* = 237, 91.2%; *p* < 0.001), diabetes (*n* = 144, 33.2% vs. *n* = 115, 44.2%), dyslipidaemia (*n* = 290, 66.8% vs. *n* = 207, 79.6%; *p* < 0.001) and chronic kidney disease (*n* = 288, 66.4% vs. *n* = 200, 76.9%; *p* = 0.003). Furthermore, patients with DA were more often smokers (*n* = 95, 21.9% vs. *n* = 88, 33.8%; *p* < 0.001). We found no differences in the use of cardiovascular medications, including betablockers, statins, platelet aggregation inhibitors (PAIs); and oral anticoagulants on baseline assessment (Table 1).

### 3.2. In-Hospital Treatment

We observed no differences between the groups concerning the frequency of MCS, the duration of ventilatory support, the incidence of acute HF or renal failure, the frequency of renal replacement therapy, bleeding episodes, or the need for the supplementary transfusion of blood products. However, patients in the DA group developed ischaemic stroke (*n* = 27, 6.2% vs. *n* = 37, 14.2% in DA; *p* < 0.001), haemorrhagic stroke (*n* = 10, 2.3% vs. *n* = 14, 5.4% in DA; *p* = 0.032) and septicaemia (*n* = 65, 15.0% vs. *n* = 54, 20.8%; *p* = 0.050) more frequently than their unaffected counterparts. During index hospitalisation, 59 (22.7%) patients with a previous history of DA experienced a relapse of depression, whereas new depression was documented in only 26 cases. Similarly, 29 (11.2%) patients from the DA group had a recurrence of anxiety, but the prevalence of new anxiety was much lower. The data were censored due to confidentiality regulations (Table 2). 

### 3.3. Outcome

**Figure 1:**
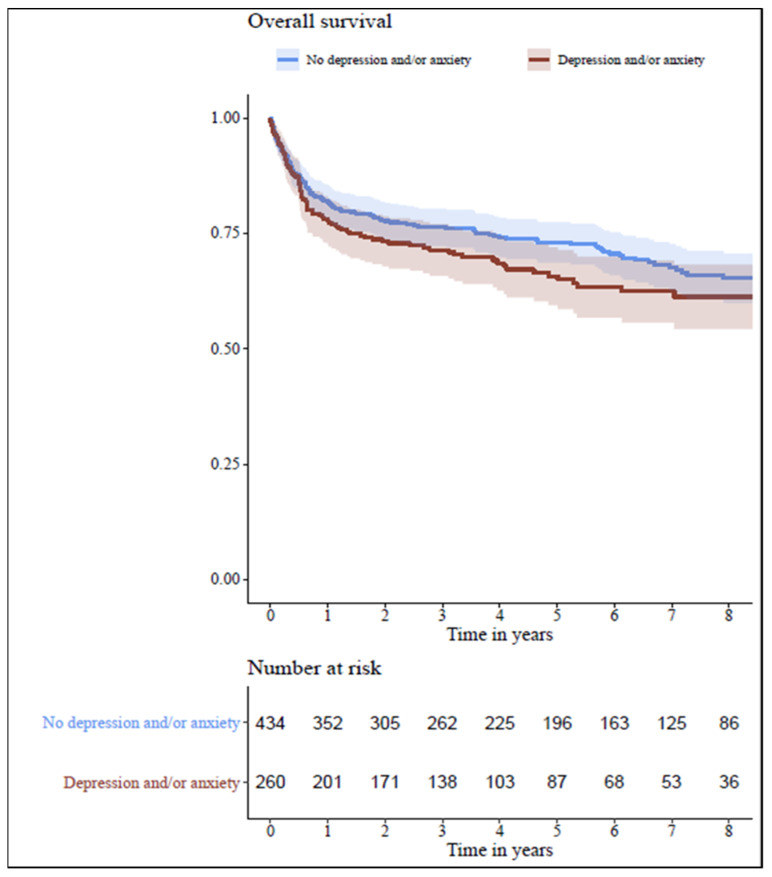
Kaplan–Meier survival estimates. Comparison of the overall survival in patients with DA (red line) and without DA (blue line) in long-term follow-up. DA—depression and anxiety.

We observed no significant differences between the two study groups with respect to the primary outcome measure (Table 2). The five-year survival rates were 65.7% and 73.1% in the groups with and without a diagnosis of DA before HTx, respectively (Figure 1). 

**Figure 2:**
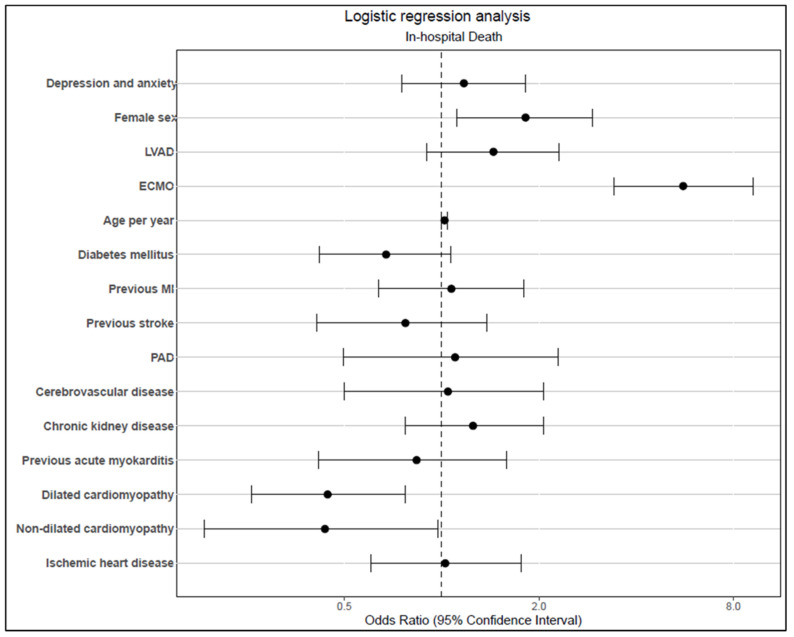
In-hospital mortality during index hospitalisation for heart transplantation. LVAD—left ventricular assist device, ECMO—extracorporeal membrane oxygenation, MI—myocardial infarction, PAD—peripheral artery disease.

However, our findings revealed that the female sex and use of extracorporeal membrane oxygenation (ECMO) during index hospitalisation were associated with inferior outcomes in a multivariate logistic regression analysis. By contrast, non-ischaemic cardiomyopathy was a determinant related to a prognostic advantage after HTx (Figure 2).

## 4. Discussion

To the best of our knowledge, this is the first study to examine the impact of DA on the outcome of HTx recipients in a relatively large patient cohort. 

Approximately one-third of the patients enrolled in our study were diagnosed with DA before HTx, which is more than previously reported [5]. One possible explanation for the increased prevalence of these disorders may relate to the modality of data acquisition. Both disorders were considered for our analyses as a composite entity. We recognise that the overall prevalence of DA cannot be calculated as a simple sum of the included diagnoses because patients often present with an overlap between the two conditions. Additionally, the ICD-10-GM system includes a range of disease severities, which allows us to reflect not only major DA episodes, but also milder cases. 

Women are generally underrepresented amongst HTx recipients, and they constituted approximately one-fifth of the overall population of our cohort [7]. These results may reflect the lack of awareness of heart disease in women due to their beneficial cardiovascular profile, resulting in a presentation at an older age with advanced disease and comorbidity profiles. Additionally, as previously reported, the prevalence of DA was higher amongst women (41.6%) in our study population compared to men (36.3%) [1]. However, regardless of sex, the disease burden by far exceeded the magnitude in the general population. 

### 4.1. Pretransplant Diagnoses and CVRF 

The prevalence of DA was higher in patients with typical cardiovascular morbidity profiles, including diabetes, hypertension, dyslipidaemia and chronic kidney disease. Previous studies have reported a bidirectional relationship between chronic diseases and mental health [2,8]. DA can add to the burden of physical illnesses, as an unhealthy lifestyle and non-adherence to prescribed medications may further accelerate the progression of the underlying conditions [9,10]. In contrast, the perception of physical illness and related concerns are associated with a higher DA prevalence [11]. Interestingly, the risk of developing mental health problems has been reported to differ based on specific patterns of physical morbidity. Thus, there are discrepancies when determining the risk of developing DA even amongst patients with cardiometabolic and cardio/cerebrovascular profiles [8]. Additionally, although active tobacco smoking during the previous six months is considered a relative contraindication for HTx, the prevalence of smoking was 11% higher amongst patients with DA in the two years preceding HTx [12]. The evidence in this field supports the notion that the risk of becoming a smoker and the daily amount of smoking is higher in people with DA, whereas the likelihood of quitting is lower than in the general population [13,14]. This may be due to the reciprocal relationship between daily habits and mental health, as smoking is usually associated with a higher risk of developing depression in addition to the bidirectional relationship between the diseases of the body and mind [15].

The higher rate of ischaemic heart disease (IHD), myocardial infarction and stroke amongst HTx recipients with DA may also be a consequence of this population’s higher cardiovascular risk profile. Previous reports have defined numerous physiologic links between DA and IHD, including dysregulation of the autonomic nervous system and peripheral vascular function, increased sympathetic tone, inflammatory activity, elevated heart rate, and endothelial dysfunction [16]. Similarly, although the overall prevalence of DA in patients with HF is higher than in the general population, differences between non-ischaemic cardiomyopathy and IHD have been reported. Particularly, patients hospitalised for acute myocardial infarction are considered to be at a high risk for major DA [17].

Although the burden of DA in patients with cancer is generally considered to be higher than in other diseases, we observed no differences in the prevalence of malignancies between the study groups [18]. This may be a consequence of the preselection of the patients, as the current recommendations for being added to the HTx waiting list take into account disease curability and require additional remission time, dependent on the type of cancer, before patients are considered for transplantation. Similarly, symptomatic CVD, PAD and severe obesity are relative contraindications for HTx [12]. This may result in a preselection of patients free from disease or only milder cases.

The prevalence of DA increases along with a prolonged time on the HTx waiting list [19]. This may explain the higher rate of previous listings in the DA group. In a world with an increasing prevalence of HF and a shortage of donor organs, awaiting HTx will be associated with a prolonged waiting time and an increased likelihood of death, which can further augment the burden on the patients’ mental health. Thus, closer monitoring and interdisciplinary assessment while being listed for HTx are required. 

### 4.2. In-Hospital Treatment

The incidence of ischaemic or haemorrhagic stroke following HTx was higher in patients with DA, despite a comparable rate of prescription of anticoagulants and PAIs. However, it is critical to recognise that antidepressants and SSRIs in particular, which are widely prescribed for patients with DA and HF because of their limited cardiovascular side effects, can cause major bleeding and ischaemic events [20,21]. Possible explanations for the increased bleeding rate are their fibrinolytic properties and potential to reduce platelet adhesion. On the contrary, ischaemic events are linked to additional factors, including limited physical activity [22]. Caution is required when prescribing SSRIs with anticoagulants, as this may result in an augmented bleeding risk. A similar association was observed in antiplatelet therapy, where SSRI prescription was previously reported to increase the risk of bleeding by 42% when prescribed in addition to aspirin and by 57% in patients on a dual antiplatelet therapy [23,24]. It is essential to investigate the association between the use of SSRIs and bleeding in the population of patients undergoing HTx. Unfortunately, we did not have access to data regarding psychotropic medication use from the patients. Due to the modality of our study, we had permission to analyse only data related to the use of cardiac drugs and anticoagulants.

Results from antecedent research indicated an increased prevalence of infectious complications and septicaemia in patients with DA who were undergoing HTx [5]. On the one hand, these findings may be linked to inflammation, but on the other hand, the downregulation of the immune system in chronic DA may also contribute to the disease course [25]. As our report focuses on in-hospital treatment and outcomes, no direct relation between these findings and a sedentary lifestyle or fidelity to taking the prescribed medication can be suspected.

### 4.3. Relapse or Post-Transplant DA

Interestingly, a previous history of DA was associated with a greater risk for relapse during index hospitalisation. By contrast, the incidence of new DA was much lower. This evidence indicates the need for a thorough pretransplant assessment so that the medical team will be aware of the patient’s condition after HTx.

### 4.4. Outcome

The five-year survival rate of the overall population was 70.4%, which is comparable with results to date [26]. Patients with DA had a slightly higher immediate post-transplant mortality, but it was not statistically significant. This can be due to the modality of our analyses, as we reported on in-hospital treatment and mortality in the immediate post-transplant period, whereas DA are more likely to have a long-term impact on a patient’s QoL. The favourable prognosis in non-ischaemic cardiomyopathy may be due to the comorbidity profile of the patients. IHD was previously reported to be associated with a significantly inferior outcome in short- and long-term follow-up [27].

The female sex and ECMO support during index hospitalisation were both associated with a significantly inferior prognosis. We should note that the current available data on sex differences and outcomes after HTx are contradictory. According to recent registry reports, female patients have a superior overall survival than men [28]. Observational studies in smaller cohorts have indicated that many other related factors such as graft size and sex mismatch may be the factors influencing the patient’s prognosis [29]. Due to the modality of our analyses, we cannot take into account these determinants. 

ECMO therapy during index hospitalisation was also a factor associated with an inferior prognosis. We cannot differentiate between pre- or post-transplant short-term MCS, which limits the value of the findings. Additionally, there were no differences in the length of hospital stay between the groups, which may rule out persisting hemodynamic instability due to sustained graft failure [30,31].

### 4.5. Strengths and Limitations

One drawback of our study is its retrospective design and the use of administrative data, which can carry a risk of a selection bias or potential confounding factors outside the scope of our analyses. The retrieved data provide an opportunity to conduct analyses focused on outcome and major complications in large cohorts. Additionally, the use of ICD codes makes it possible to cover a range of disease severities and to reflect not only major DA episodes, but also milder cases. However, to reduce potential bias, the study findings still need to be investigated in a randomised manner.

Another drawback of this approach is the limited insight into the patients’ clinical condition aside from the diagnoses. We report on the recovery rate, but we cannot take into account the grade of recovery (exercise capacity and NYHA class) after HTx. Additionally, some factors such as size and sex mismatch, donors’ characteristics and recipients’ laboratory results, which may influence the outcome of the patients, cannot be assessed.

## 5. Conclusions

DA are commonly diagnosed in patients undergoing HTx, particularly in those with multiple comorbidities. This underscores the bidirectional association between the disease of the body and the mind. Whilst we observed a higher prevalence of ischaemic stroke, haemorrhagic stroke and septicaemia in the immediate post-transplant period in patients diagnosed with DA, the latter had no prognostic relevance in long-term follow-up. Additionally, the incidence of new DA was lower than the rate of relapse in patients already diagnosed with these conditions. This observation suggests that pretransplant DA may set the stage for recurrent mental health disorders following HTx. Therefore, closer monitoring of these patients during the post-transplant phase is crucial to the success of the treatment mission.

## Figures and Tables

**Table 1:** Baseline characteristics.

Characteristics	Non-DA *n* = 434	DA *n* = 260	Study Population *n* = 694	*p*-Value
Female, *n* (%)	90 (20.7)	64 (24.6)	154 (22.2)	0.234
Male, *n* (%)	344 (79.3)	196 (75.4)	540 (77.8)	0.234
VAD, *n* (%)	105 (24.2)	70 (26.9)	175 (25.2)	0.423
Age, median (IQR)	52.52 (14.53)	54.28(11.70)	53.12 (13.56)	0.053
Previous acute myocarditis, *n* (%)	56 (12.9)	34 (13.1)	90 (13.0)	0.947
Dilated cardiomyopathy, *n* (%)	325 (74.9)	195 (75.0)	520 (74.9)	0.973
Non-dilated cardiomyopathy, *n* (%)	44 (10.1)	23 (8.8)	67 (9.7)	0.577
Ischaemic heart disease, *n* (%)	276 (63.6)	200 (76.9)	476 (68.6)	<0.001
Hypertension, *n* (%)	356 (82.0)	237 (91.2)	593 (85.4)	<0.001
Diabetes, *n* (%)	144 (33.2)	115 (44.2)	259 (37.3)	0.004
Dyslipidaemia, *n* (%)	290 (66.8)	207 (79.6)	497 (71.6)	<0.001
Obesity, *n* (%)	127 (29.3)	86 (33.1)	213 (30.7)	0.292
Smoking, *n* (%)	95 (21.9)	88 (33.8)	183 (26.4)	<0.001
Atrial flutter/fibrillation, *n* (%)	297 (68.4)	177 (68.1)	474 (68.3)	0.922
PAD, *n* (%)	30 (6.9)	23 (8.8)	53 (7.6)	0.353
CVD, *n* (%)	40 (9.2)	26 (10.0)	66 (9.5)	0.733
Chronic kidney disease, *n* (%)	288 (66.4)	200 (76.9)	488 (70.3)	0.003
Cancer, *n* (%)	44 (10.1)	24 (9.2)	68 (9.8)	0.697
No previous listing, *n* (%)	187 (43.1)	82 (31.5)	269 (38.8)	0.003
Previous MI, *n* (%)	129 (29.7)	108 (41.5)	237 (34.1)	0.001
Previous PCI, *n* (%)	54 (12.4)	51 (19.6)	105 (15.1)	0.011
Previous stroke, *n* (%)	54 (12.4)	55 (21.1)	109 (15.7)	0.002
PAIs, *n* (%)	51 (11.8)	34 (13.1)	85 (12.2)	0.606
OACs, *n* (%)	106 (24.4)	65 (25.0)	171 (24.6)	0.865
PAIs in combination with OACs, *n* (%)	44 (10.1)	26 (10.0)	70 (10.1)	0.953
ACE-Is/AT1-antagonists, *n* (%)	204 (47.0)	126 (48.5)	330 (47.6)	0.710
Statins, *n* (%)	109 (25.1)	67 (25.8)	176 (25.4)	0.848
Betablockers, *n* (%)	252 (58.1)	153 (58.8)	405 (58.4)	0.840

Data are presented as number (percentage) or median (IQR). DA—depression and anxiety, VAD—ventricular assist device, PAD—peripheral artery disease, CVD—cerebrovascular disease, PCI—percutaneous coronary intervention, MI—myocardial infarction, PAIs—platelet aggregation inhibitors, OACs—oral anticoagulants, ACE-Is—angiotensin-converting enzyme inhibitors, AT1-antagonists—angiotensin 1 receptor antagonists.

**Table 2:** Outcome.

Characteristics	Non-DA *n* = 434	DA *n* = 260	Overall Population *n* = 694	*p*-Value
ECMO, *n* (%)	65 (15.0)	34 (13.1)	99 (14.3)	0.488
Acute renal failure, *n* (%)	184 (42.4)	107 (41.2)	291 (41.9)	0.748
Renal replacement therapy, *n* (%)	287 (66.1)	173 (66.5)	460 (66.3)	0.912
Death (discharge status), *n* (%)	62 (14.3)	42 (16.2)	104 (15.0)	0.505
Ischaemic stroke, *n* (%)	27 (6.2)	37 (14.2)	64 (9.2)	<0.001
Haemorrhagic stroke, *n* (%)	10 (2.3)	14 (5.4)	24 (3.5)	0.032
Bleeding, *n* (%)	172 (39.6)	106 (40.8)	278 (40.1)	0.767
Ventilation, median (IQR)	71 (279)	66 (306)	70 (289)	0.933
Hospitalisation, median (IQR)	129 (130.3)	137 (142.8)	133 (137.0)	0.511
In-hospital CPR, *n* (%)	63 (14.5)	33 (12.7)	96 (13.8)	0.501
Blood transfusion, *n* (%)	393 (90.6)	234 (90.0)	627 (90.3)	0.811
Septicaemia, *n* (%)	65 (15.0)	54 (20.8)	119 (17.1)	0.050
Allograft rejection, *n* (%)	88 (20.3)	58 (22.3)	146 (21.0)	0.525
New depression, *n* (%)	26 (6.0)	N/A	26 (3.7)	N/A
Depression relapse, *n* (%)	N/A	59 (22.7)	59 (8.5)	N/A
New anxiety, *n* (%)	<10	N/A	N/A	N/A
Anxiety relapse, *n* (%)	N/A	29 (11.2)	29 (4.2)	N/A
PAIs	36 (10.0)	25 (11.9)	61 (10.7)	0.478
OACs, *n* (%)	<10	<10	N/A	0.740
PAIs in combination with OACs, *n* (%)	<10	<10	N/A	0.190
ACE-Is/AT-blockers, *n* (%)	41 (11.4)	27 (12.9)	68 (11.9)	0.602
Statins, *n* (%)	59 (16.4)	46 (21.9)	105 (18.4)	0.101
Betablockers, *n* (%)	14 (3.9)	15 (7.1)	29 (5.1)	0.088

Data are presented as number (percentage) or median (IQR). DA—depression and anxiety, ECMO—extracorporeal membrane oxygenation, PAIs—platelet aggregation inhibitors, OACs—oral anticoagulants, ACE-Is—angiotensin-converting enzyme inhibitors, AT1-antagonists—angiotensin 1 receptor antagonists. N/A—not applicable.

## Data Availability

The authors confirm that the data utilised in this study cannot be made available in the manuscript, in the Supplementary Files or in a public repository due to German data protection laws (‘Bundesdatenschutzgesetz’, BDSG). Therefore, they are stored on a secure drive in the AOK Research Institute (WIdO), to facilitate replication of the results. Generally, access to data of statutory health insurance funds for research purposes is possible only under the conditions defined in German Social Law (SGB V § 287). Requests for data access can be sent as a formal proposal specifying the recipient and purpose of the data transfer to the appropriate data protection agency. Access to the data used in this study can only be provided to external parties under the conditions of a cooperation contract with this research project and after written approval by the sickness fund. For assistance in obtaining access to the data, please contact wido@wido.bv.aok.de.

## Supplementary Materials

The following supporting information can be downloaded at: https://www.mdpi.com/article/10.3390/jpm13050844/s1, Supplementary Tables S1 and S2: Diagnoses and procedural codes; Table S3: In-hospital mortality during index hospitalisation for heart transplantation.
