# Supplementary material for: Depression and Anxiety in Heart Transplant Recipients: Prevalence and Impact on Post-Transplant Outcomes
Source: J Pers Med. 2023 May 17;13(5):844. doi: 10.3390/jpm13050844 (PMC10220721; doi:10.3390/jpm13050844)
Supplement: Supplementary file 1 [file jpm-13-00844-s001.zip › jpm-2375688-supplementary.pdf]

Supplementary Material

Table S1. Diagnoses and procedural codes.

Diagnoses, Procedures and Medical Treatment	Corresponding Codes
VAD	5-376.20, 5-376.22, 5-376.23, 5-376.30, 5-376.33, 5-376.34, 5-376.40, 5-376.50, 5-376.60, 5-376.70, 5-376.72, 5-376.80, 5-376.83, 5-376.84, 5-376.90, 5-376.91, 5-376.92, 5-376.93
Acute myocarditis	I40, I41, I51.4
Dilated cardiomyopathy	I42.0
Non-dilated cardiomyopathy	I42.1, I42.2, I42.3, I42.4, I42.5, I42.6, I42.7, I42.8, I42.9, I43.0, I43.1, I43.2, I43.8
Ischemic heart disease	I25
Hypertension	I10 – I15
Diabetes mellitus	E10 – E14
Dyslipidemia	E78
Obesity	E66
Smoking	F17
Atrial flutter/fibrillation	I48
PAD	I70.20, I70.21, I72.22, I72.23, I72.24, I72.25, I72.29
CVD	I67.2
Chronic kidney disease	N18, N19
Cancer	C
Previous listing	1-920.01, 1-920.21, 1-920.31, U55.10, U55.11, U55.12
Previous myocardial infarction	I21, I22
Previous PCI	8-837

Previous Stroke	I60 - I64, I69
PAI	B01AC04, B01AC06, B01AC22, B01AC24
OAC	B01AF01 - B01AF04, B01AA04
ACE-I or AT1-antagonists	C01AA, C09C
Statins	C10AA01 – C10AA08
Betablocker	C07

Diagnoses were coded according to the German Modification of the International Statistical Classification of Diseases and Related Health Problems 10th Revision (ICD-10-GM). Procedures were coded according to the German Procedure Classification (OPS). Anatomical Therapeutic Chemical codes (ATC-codes) were used for the prescribed medication.

Abbreviations: DA – depression and anxiety, VAD – ventricular assist device, PAD – peripheral artery disease, CVD – cerebrovascular disease, PCI - percutaneous coronary intervention, PAI - platelet aggregation inhibitor, OAC – oral anticoagulants, ACE-I - angiotensin-converting enzyme inhibitors, AT1-antagonists – angiotensin 1 receptor antagonists

Table S2. Diagnoses and procedural codes.

Diagnoses, Procedures and Medication	Corresponding Codes
ECMO	8-852.3
Acute renal failure	N17
Renal replacement therapy	8-853 – 8-855, 8-857
Ischemic stroke	I63, I64
Haemorrhagic stroke	I60 – I62
Bleeding	K92, H44.8, T81.0, T81.2, T81.3, T81.7
Ventilation	8-713
In-hospital CPR	8-771
Blood transfusion	8-800.C
Septicaemia	A41
Allograft rejection	T86.2
Depression	F30 – F39
Anxiety	F41

Diagnoses were coded according to the German Modification of the International Statistical Classification of Diseases and Related Health Problems 10th Revision (ICD-10-GM). Procedures were coded according to the German Procedure Classification (OPS). Anatomical Therapeutic Chemical codes (ATC-codes) were used for the prescribed medication.

Abbreviations: ECMO - extracorporeal membrane oxygenation, CPR - cardiopulmonary resuscitation.

Table S3. In-hospital mortality during index hospitalisation for heart transplantation.

**In-Hospital Mortality**			
Variables	Hazard ratio	95% confidence interval	p value
Depression and anxiety	1.17	0.75 – 1.82	0.48
Female gender	1.82	1.12 – 2.93	0.02
LVAD prior to HTx	1.45	0.90 – 2.30	0.12
ECMO prior to HTx	5.59	3.41 – 0.91	1.10
Age per year	1.02	1.00 – 1.04	0.07
Diabetes mellitus	0.67	0.42 – 1.10	0.10
Previous MI	1.10	0.64 – 1.79	0.79
Previous stroke	0.77	0.41 – 1.38	0.40
PAD	1.10	0.50 – 2.29	0.80
Cerebrovascular disease	1.04	0.50 – 2.10	0.90
Chronic kidney disease	1.25	0.77 – 2.10	0.37
Previous acute myocarditis	0.84	0.42 – 1.60	0.60
Dilated cardiomyopathy	0.44	0.26 – 0.77	0.004
Non-dilated cardiomyopathy	0.44	0.18 – 0.97	0.05
Ischemic heart disease	1.03	0.60 – 1.76	0.93

HTx – heart transplantation, LVAD – left ventricular assist device, ECMO - Extracorporeal Membrane Oxygenation, MI – myocardial infarction, PAD – peripheral artery disease
